# Isolated tricuspid valve endocarditis - A rare entity and a surgeon’s dilemma

**DOI:** 10.34172/jcvtr.2022.13

**Published:** 2022-06-12

**Authors:** Rahul Bhushan, Vaibhav Chugh, Narender S Jhajhria, Vijay Grover, Palash V Aiyer

**Affiliations:** Department of CTVS, ABVIMS and Dr RML Hospital, New Delhi, India

**Keywords:** Tricuspid Valve Endocarditis, Vegetectomy, Patch Repair

## Abstract

Isolated tricuspid valve endocarditis accounts for only 5 to 10 percent of infective endocarditis cases globally. Numerous surgical procedures ranging from simple vegetectomy, creation of neoleaflets or complete replacement by a prosthetic valve have been described. We aimed to evaluate our experience in surgical management of this entity and to formulate an approach for timing, appropriateness and extent of surgery. Patients operated on semi elective/emergency basis had adverse outcome with residual regurgitation and had longer ICU stay. Also, patients who required excision of leaflet and creation of neoleaflets had a higher incidence of regurgitation. This suggests that maximal preservation of native valve lessens the incidence of residual regurgitation. Simple vegetectomy and patch repair of the residual defect offers the best outcome.

## Introduction

 Isolated tricuspid valve (TV) endocarditis accounts for only 5 to 10 percent of infective endocarditis cases globally.^1^ It is even rarer in the Indian scenario owing to the lesser incidence of intravenous drug abusers and pacemaker implantations as compared to west.^2^ In a prospective study carried out by Gupta et al^2^ only one patient had vegetation over the tricuspid valve out of 61 patients of infective endocarditis studied. Numerous surgical procedures ranging from simple vegetectomy, creation of neoleaflets following excision of affected leaflet to complete excision of the native valve and replacement by a prosthetic valve have been described.^3^ The purpose of our study was to evaluate our centers` experience in the surgical management of this entity and to formulate an approach for timing, appropriateness and extent of surgery for this rare disease.

## Materials and Methods

 This is a retrospective study carried out at ABVIMS and Dr RML hospital, a tertiary care centre in New Delhi, India. A total of 6 patients were operated for this disease from April 2019 to April 2021. Detailed preoperative history taking and evaluation was done in all cases to evaluate any intracardiac shunt, peripheral abscesses, osteomyelitis, and any IV drug abuse to assess risk factors for TV endocarditis.

 Out of the six patients operated, four patients were electively operated after adequate and appropriate antibiotic therapy once they were culture negative and afebrile. Indication for surgery in these patients was clinical evidence of right heart dysfunction and Echocardiographic finding of severe Tricuspid valve regurgitation (TR) along with vegetation.

 Two patients were operated on a semi elective basis due to large size of the vegetation, progressive heart failure despite medical management and recurrent lung embolization from the vegetation.

 All patients were kept on serial clinical and echocardiography (ECHO) follow-up – weekly for one month and three monthly thereafter.

###  Surgical Technique

 Vegetectomy was carried out in all cases with adequate margin to ensure removal of all infective foci. The valve was reevaluated after vegetectomy and appropriate reconstructive technique was planned. Small residual defects in the leaflets were closed with autologous untreated pericardial patch using 5 0 polypropylene sutures. In patients where the whole leaflet was excised, neoleaflet was constructed using autologous pericardium. Residual tricuspid regurgitation was evaluated by saline insufflations during cardioplegic arrest and by transesophageal echocardiography after removal of cross clamp. Residual tricuspid regurgitation was managed by limited annuloplasty using autologous pericardium. Once satisfactory leaflet coaptation and tricuspid repair was achieved, right atrium was closed in a single layer and patient was weaned of cardiopulmonary bypass

## Results

 Vegetations were multiple in most cases (Table 1) and varied from few mm to 2 cm in size. Most common organism on blood culture was Staphylococcus Aureus seen in 4 patients, 1 patient had culture report of Enteroccci while culture of 1 patient came out to be sterile.

**Table 1:** Demography, evaluation and intervention data

**Patient Number**	**Demography of patient **	**Vegetation morphology and size **	**Degree of TR** **(Preoperative) (ECHO)**	**Blood Culture**	**Timing of intervention**	**Procedure**
1	34 years, female	Anterior leaflet, 1.2cms	Severe TR	Staphylococcus Aureus	Elective	Vegectectomy+ patch Repair
2	37 years, female	Antero septal leaflet, 8mm	Severe TR	Staphylococcus Aureus	Elective	Vegectectomy+ patch Repair
3	9 years, female	All 3 leaflets (5mm, 1.6cm, 9mm)	Severe TR	Staphylococcus Aureus	Elective	Neoleaflet creation
4	9 years, female	Anterior and septal leaflets (1.6cm, 5mm)	Moderate TR	Enterococcus	Semi Urgent (pulmonary embolization and right heart failure)	Vegectectomy+ patch Repair
5	27 years, male	All 3 leaflets (5mm, 1.7cm, 9mm)	Severe TR	Sterile	Elective	Neoleaflet creation
6	29 years, female	All 3 leaflets (2.1cm, 6mm, 8mm)	Severe TR	Staphylococcus Aureus	Semi Urgent (pulmonary embolization)	Neoleaflet creation

 All patients were evaluated by transesophageal (TEE) echo intraoperatively for adequacy of repair and degree of residual TR if any and followed up by serial transthoracic (TTE) echocardiography in the post op period for residual disease or progression of disease.

 While four patients on TEE intraoperatively had mild TR, two patients had moderate TR with central jet which was acceptable without any evidence of right ventricular dysfunction and acceptable hemodynamics. Both these patients belonged to surgical group which were taken on semi urgent basis for surgical repair. They also required inotropic support along with decongestive measures in the postoperative period. These patients were taken up for surgery with elevated inflammatory markers in view of their symptomatic status despite optimal medical therapy.

 Subsequently, one other patient had progression of severity of TR from mild to moderate in intensity after one month follow up in post operative period. The symptoms were successfully managed by diuretics and fluid restriction and needed no further surgical intervention. This patient had severe TR preoperatively and had to undergo leaflet reconstruction of all three leaflets following wide excision of the vegetation.

 None of the patients in our study had any conduction block requiring pacemaker insertion and there was no mortality amongst the operated patients. No patient underwent prosthetic valve implantation in our series.

## Discussion

 Surgical intervention in tricuspid endocarditis remains an enigma for surgeons regarding the timing and type of intervention. Medical management with appropriate antibiotics and decongestive therapy remain the cornerstone of therapy for this disease entity. Isolated tricuspid endocarditis follows a more benign course as compared to left sided endocarditis involving the aortic and mitral valve.^4^

 While initial consensus was that tricuspid valve endocarditis responds well to conventional antibiotic therapy, long term follow up suggested that progression to heart failure was high with mortality rate ranging from 50 to 90 percent in such cases.^5^

 In his pioneer work Hughes et al suggested that, where indicated, early operation and vegetectomy may have even greater benefits than have been previously appreciated, including preservation of the normal, native valve, preservation of normal hemodynamic function, abolition of the risks of anticoagulative medication, and abolition of the risks of prosthetic endocarditis.^5^ This paved way for simple vegetectomy as a viable surgical option in complete treatment of this entity.

 There are limited surgical options due to the inherent complexity of the mechanics of the tricuspid valve and persistence of mild to moderate regurgitation after repair.^6^However, residual regurgitation of the tricuspid valve is well tolerated and easily managed medically.^6^ Historically, Arbulu et al proposed tricuspid valve excision without replacement as adequate treatment, highlighting that tricuspid valve regurgitation is well tolerated.^7^ Complete excision of all infected tissue and restoration of valvular competence is the cornerstone of surgical management. Preservation of native tissue and valve reconstructive procedures such as leaflet enhancement using autologous pericardium and limited annuloplasty assist in achieving this aim.^8^ However, there is some risk of recurrence of infection and progressive valvular dysfunction with this approach.^9^ Replacing the tricuspid valve with a prosthetic valve is an option for severely diseased valves where preservation of native tissue is not possible.^10^ However, they have their own inherent disadvantages – anticoagulation in mechanical prosthesis and degeneration in bioprosthetic valves.^11^

 In our series, two patients were taken up for surgical correction on a semi elective basis and had elevated inflammatory markers. These patients had residual regurgitation and had longer ICU stay.^12^ This reiterates that adequate clearance of infection by optimal antibiotic therapy should always be the cornerstone of therapy^12^. Also, patients who required excision of leaflet and creation of neoleaflets had a higher incidence of regurgitation. This suggests that maximal preservation of native valve lessens the incidence of residual regurgitation. Simple vegetectomy and patch repair of the residual defect offers the best outcome. However, this technique also requires meticulous excision of all infected tissue and a certain amount of surgical skill.

 The limitation of our series was the small number of patients and no long term follow-up.

## Conclusion

 To conclude, optimum medical management with appropriate antibiotics forms the first line of management of isolated tricuspid valve endocarditis before consideration of surgical intervention. Simple vegetectomy and patch repair of the residual defect offers simple and effective outcome in patients who are optimized medically. Asymptomatic mild to moderate tricuspid regurgitation in patients who had severe regurgitation preoperatively was well tolerated. However, this group of patients requires longer follow up to assess further progression of disease.

## Acknowledgments

 The authors would like to thank the co-authors, teachers and colleagues who have contributed at various stages through the development of this publication.

## Funding

 The project did not receive any funding.

## Ethical approval

 The study was approved by the ethical committee of ABVIMS and Dr RML Hospital, New Delhi (code: 550(86/2021)/IEC/ABVIMS/RMLH/698).

## Competing interest

 The authors declare that there are no conflicts of interest.
